# Microbiome Associated with the Mycangia of Female and Male Adults of the Ambrosia Beetle *Platypus cylindrus* Fab. (Coleoptera: Curculionidae)

**DOI:** 10.3390/insects12100881

**Published:** 2021-09-29

**Authors:** Stefano Nones, Fernanda Simões, Cândida Sofia Trindade, José Matos, Edmundo Sousa

**Affiliations:** 1 Agrarian and Forestry Systems and Vegetal Health Unit, Instituto Nacional de Investigação Agrária e Veterinária (INIAV), Quinta do Marquês, 2780-159 Oeiras, Portugal; candida.trindade@iniav.pt (C.S.T.); edmundo.sousa@iniav.pt (E.S.); 2 GREEN-IT Bioresources for Sustainability, ITQB NOVA, Quinta do Marquês, 2780-157 Oeiras, Portugal; 3 Instituto de Tecnologia Química e Biológica António Xavier, Universidade Nova de Lisboa, Quinta do Marquês, 2780-157 Oeiras, Portugal; 4 Biotechnology and Genetic Resources Unit, Instituto Nacional de Investigação Agrária e Veterinária (INIAV), Quinta do Marquês, 2780-159 Oeiras, Portugal; fernanda.simoes@iniav.pt (F.S.); jose.matos@iniav.pt (J.M.); 5 Centre for Ecology, Evolution and Environmental Changes, Faculdade de Ciências, Universidade de Lisboa, Campo Grande, 1749-016 Lisboa, Portugal

**Keywords:** sexual dimorphism, metabarcoding, Platypodinae, prokaryotes, cork oak

## Abstract

**Simple Summary:**

The ambrosia beetle *Platypus cylindrus* is a major cork oak pest in Portugal. Beetles have different roles in host tree colonization and are equipped with specific structures (mycangia) for fungal transportation. The information on bacterial composition associated with mycangia is scarce. The bacterial community present in the mycangia of *P. cylindrus* male and female beetles collected from cork oak galleries was investigated. Mycangia anatomical structure was also explored using histological and 3D imaging techniques to highlight evidence of biological sexual dimorphism. A diverse bacterial community with few gender-specific bacteria was shown and histology revealed connections linking external and internal tissues only in females, providing the first insights into sexual differentiation for bacteria in a Platypodinae beetle species.

**Abstract:**

The ambrosia beetle *Platypus cylindrus* Fab. (Coleoptera: Curculionidae) is a major cork oak pest in Portugal. Female and male beetles have different roles in host tree colonization and are both equipped with prothoracic mycangia for fungal transportation. Despite a known beneficial role of bacteria in ambrosia beetles, information on bacterial composition associated with prothoracic mycangia structures is scarce. Bacterial community from mycangia of *P. cylindrus* male and female beetles collected from cork oak galleries was investigated by means of 16S metagenomics. Mycangia anatomical structure was also explored with histological techniques and X-ray computed microtomography to highlight evidence supporting biological sexual dimorphism. A bacterial community with highly diverse bacterial taxa with low abundances at the genus level was revealed. Lactobacillales, *Leptotrichia*, *Neisseria*, *Rothia*, and Sphingomonadaceae were significantly more abundant in males, while *Acinetobacter*, Chitinophagaceae, Enterobacteriaceae, Erwiniaceae, Microbacteriaceae, and *Pseudoclavibacter* were more abundant in females. Additionally, a core bacteriome of five genera was shared by both sexes. Histological examination revealed visible connections linking external and internal tissues in females, but none in males. Overall, these results provide the first insights into sexual differentiation for bacteria in a Platypodinae beetle species, identifying key patterns of bacteria distribution in the context of beetle ecology and functional behavior.

## 1. Introduction

Ambrosia beetles (Coleoptera: Curculionidae: Scolytinae and Platypodinae) are an ecological assemblage of wood-boring weevils, originated from the independent evolution of different clades, largely within bark beetles. The dominant ecological strategy of ambrosia beetles is based on fungus farming in trees [1]. This represents the most ancient agricultural system of insects, originated within the Platypodinae subfamily, in the mid-Cretaceous (~96 Ma) [2]. Ambrosia beetles form obligate nutritional symbioses with the fungi that grow across the natal galleries bored into the xylem of host trees, essential for larval feeding. The adults carry the fungi, some of them phytopathogenic, from tree to tree in specialized structures called mycangia [3]. The mycangia of beetles from Platypodinae correspond to an ovoidal membranous invagination, located on the flat middle upper part of the prothorax [4]. Mycangia offer a protected environment for the fungal symbionts. These structures feature low exposure to UV light, abrasion, and plant metabolites, as well as provide nutrients and support the selection of specific microbes [3].

The presence of bacteria in the mycangia of ambrosia beetles was previously reported [3], including species-specific strains potentially contributing to the selection of specific ambrosia fungi [5]. Recently, the role of bacteria in the ecology of the ambrosia beetle *Xyleborus affinis* has been updated from a new metagenomic analysis suggesting a central role in wood degradation and nutrient supplementation for fungi and beetles [6,7]. Information on possible bacterial differentiation according to the gender of the associated beetle is scarce and it would suggest homogeneous bacterial diversity. However, a few differences at the genus level were reported in feces and guts of bark beetle species [8,9].

The oak pinhole borer *Platypus cylindrus* Fab. (Coleoptera: Curculionidae) is a species distributed across Eurasia and the Mediterranean area. It became notorious in Portugal in the last decades after its shift of ecological habit, as it exchanged from infesting decaying trees to promoting tree death in asymptomatic cork oaks (*Quercus suber* L.) in just a few years [10]. The associated fungi are well-studied and some are proposed as declining agents [11,12]. However, little information exists about the bacteria of Platypodinae beetles [13]. To date, only the studies of Nones et al. [13] and Tarno et al. [14] report the isolation of bacterial strains from mycangia of *P. cylindrus* in Portugal and of *Euplatypus parallelus* in Indonesia (i.e., *Streptomyces* sp.), respectively.

Sex differentiation is important in the ecology of *P. cylindrus*. Female and male adults possess different mycangial structures and play a key diverse role in the colonization of trees [10]. The female mycangium is about three times bigger than that of the males. Moreover, it possesses hundreds of integumentary pits harboring fungal spores, divided into two groups by a straight cuticular line. Conversely, the male mycangium has less than a dozen pits, arranged on the cuticle like in the female [4]. 

The establishment of the insects on a host is led primarily by male adults that select the host tree, attract other males by releasing an aggregation pheromone, and excavate penetration corridors in the tree trunk. Afterwards, the coupling ritual starts and ends with females leading the construction of the deepest tract of the galleries, laying eggs, and spreading fungal spores. At this point, the males are only involved in sawdust removal and gallery entrance protection. The offspring develops inside the galleries, emerging as adults from the entry holes made by the parents [10].

The knowledge about *P. cylindrus* gender behavior, fungal symbiosis, life cycle, and deleterious effect on cork oak stands led us to focus on questions regarding the bacterial role and community composition in the male and female mycangia of *P. cylindrus* and respective ecological implications. Therefore, we studied the microbiota of male and female *P. cylindrus* beetles emerged from the trunk of cork oaks using high-throughput sequencing. Moreover, we explored the anatomical structure of the mycangia with histological techniques and X-ray computed microtomography to highlight novel traits supporting biological sexual dimorphism.

## 2. Materials and Methods

### 2.1. Beetle Mycangia Sampling

A total of five cork oak trees (T1–T5) infested by *Platypus cylindrus*, of similar age (50 years old) and size (diameter at breast height = 120 ± 16 cm), were selected from a Portuguese cork oak stand in Alentejo (Portugal). The trees were obtained in winter 2018 and were located up to 500 m from each other. They were cut down, divided into logs, and brought to the lab, where they were maintained at a constant temperature and relative humidity (T = 18 °C; R.H. = 70%). The upper and lower sides were covered with a layer of paraffin to preserve wood moisture. Each gallery was numbered, and a short polycarbonate tube connected to a fabric net was placed on the entrance. Beetles from three selected tree galleries were captured alive, sorted by sex, and individually rinsed with 50 µL of sterile distilled water. Beetles not immediately used were stored at −20 °C. Mycangia were excised from the body of preferentially living beetles by delimiting a rectangular shape on the cuticle with a sterile surgery scalpel under a stereoscope and immediately placed into microtubes under the hood. The remaining body parts were discarded. The mycangia were crushed altogether with a sterile pestle and incubated overnight in 500 µL of phosphate-buffered saline solution (PBS 10 mM, pH 7.2) at 6 ± 2 °C in an orbital shaker. Afterwards, the extract was stored at −70 °C until further use. In total, 90 beetles were grouped into 30 mycangia samples comprising three mycangia from beetles with the same sex and gallery.

### 2.2. DNA Extraction and Sequencing

DNA extraction from the frozen extract obtained as described above was performed using an innuSPEED Bacteria/Fungi DNA Kit (Analytik Jena, Germany), according to the manufacturer’s instructions. Two negative control extractions were performed with the kit using only an elution buffer and water. Sequencing of the V4 region of the 16S rRNA gene using the barcoded primer set 515F-806R [15] was performed at the Genomics Unit of Instituto Gulbenkian de Ciência (Oeiras, Portugal), using a 280-multiplex approach on a 2 × 250 bp PE Illumina MiSeq run. Sequence data have been deposited in the European Nucleotide Archive (ENA) at EMBL-EBI under accession number PRJEB44517: SAMEA8616474–SAMEA8616503 (https://www.ebi.ac.uk/ena/browser/view/PRJEB44517; Accessed on 28 May 2021).

### 2.3. Bioinformatic Analysis

The raw paired-end FASTQ reads were processed using the Quantitative Insights Into Microbial Ecology 2 program (QIIME2, ver. 2020.2, [16]), with default parameters. DADA2 was used for quality filtering, denoising, paired-end merging, and Amplicon Sequence Variant (ASV) calling, using the plug-in q2-dada2 with the denoise-paired method [17,18,19,20]. ASVs are a higher-resolution analogue of the traditional OTUs, resolved to the level of single-nucleotide differences [21]. Quality filtering was achieved by carrying out reads removal with a Phred quality score (Q) less than 20.5 on average. Moreover, the Naïve Bayes classifier was obtained for the V4 region following the procedure of Robeson [22] with SILVA 138 SSU NR 99 database and trained in QIIME2 with a q2-feature-classifier fit-classifier-naive-bayes [23,24]. Taxonomical classification was determined by matching ASVs against the Silva database [25] with a q2-feature-classifier classify-sklearn [23]. Removal of nonbacterial sequences from the feature table (i.e., ASV table) and ASV sequences was performed using filter methods. Singletons were not removed, as they can play an important role in the environment [26]. To visualize the taxonomic composition of the samples, a q2-taxa barplot was used. ASV sequences were aligned using MAFFT [27] and used to construct a phylogenetic tree with FastTree 2 [28]. Libraries were then rarefied to the same sequencing depth of 1260 sequences. Alpha rarefaction plots were obtained with q2-diversity, using default settings except for “steps” (25) and “max depth” (2000 and 4000) to test adequacy of sampling.

Features having only one entry across the samples and with low abundances (10 for ASVs, 20 for genus) of unrarefied data were filtered out from the tables with ASV sequences and with ASVs collapsed at the genus level (q2-taxa collapse). The “core” mycangia bacteriome was then calculated as the ASVs present in ≥55% of all samples.

### 2.4. Statistical Analysis

The phylogenetic tree and the rarefied feature table were used to calculate alpha and beta indexes incorporating phylogenetic information. Four alpha diversity indexes (observed features, Shannon, Faith’s Phylogenetic Diversity (PD), Pielou’s evenness) and four beta diversity metrics (Bray–Curtis, Jaccard, weighted and unweighted UniFrac) were calculated in QIIME2 using q2-diversity [20,29,30,31,32]. Alpha diversity was compared between the different groups of samples using the Kruskal–Wallis test. Principal coordinate analysis (PCoA) was performed based on Bray–Curtis and weighted UniFrac distances and visualized with EMPeror [33]. Data separation in the PCoA was tested using the Permutational multivariate analysis of variance (PERMANOVA), and *p*-values were generated based on 999 permutations.

For differential abundant analysis at the genus level, filtered and unrarefied ASV tables were imported into R 4.0.3 [34]. Missing imputation was performed by adding a value of one in the original data. Using the package DAtest 2.7.17 [35], an optimal statistical test was identified for the ASV tables. DAtest is a statistical suite for differential abundance analysis of “omics” data. Briefly, DAtest finds the most appropriate method for the specific dataset by repeated empirical testing. Based on the evaluation output (Supplementary Table S1), we selected the Welch’s *t*-test with centered log-ratio transform.

### 2.5. Analysis of Mycangia Anatomical Structure

A total of 13 random beetles (6 males and 7 females) were used for the preliminary exploration of the anatomical structure of the prothoracic mycangia. Samples of *P. cylindrus* beetles were fixed for at least 48 h in FAA 1:1:18 formalin/acetic acid/ethanol (70%) at 4 °C [36,37]. Dehydration was achieved through progressive acetone (100%) series. The low-viscosity epoxy resin embedding medium ERL-4206 [38] with a modified D.E.R. 736 (diglycidyl ether of polypropylene glycol) content of 6.5–7.3 g was used. The resin components were stirred for at least 20 min to remove air bubbles. For resin impregnation, the beetles were transferred from acetone into vials with a 1:1 mixture of acetone/epoxy resin, for 6 h. Vials were then opened to allow the acetone to evaporate overnight (12–15 h). Afterwards, the beetles were placed in pure resin for 1 h and then transferred to an embedding mold with fresh resin. The temperature was raised to 40 °C to reduce resin viscosity. This procedure was repeated, and any adhering air bubbles were removed using a needle. Complete polymerization took place in an oven at 70 °C within 24 h. Histological sections (female and male mycangia) of 2 μm thickness were obtained using a rotary microtome Leica RM2255 (Leica, Germany) and stained with Cotton and Astra blue + safranin. Image acquisition was performed using a Digital Microscope Leica© DMS1000 with LAS (Leica Application Suite) V4.4 software.

Microcomputed tomography (µ-CT) was used as a 3D microscope to visualize the structure of the prothorax. Samples of fresh, dead, and intact beetle bodies were selected and conditioned in the refrigerator at a temperature of 10 °C. The samples were scanned using an X-ray µ-CT scanner Skyscan 1172 with a VDS 1.3 Mp camera (Bruker Instruments, Inc., San Jose, CA, USA). To optimize details and enhance contrast, 1.81 µm image pixel size with 48 kV source voltage and 208 µA source current were used (10 W power). Samples were imaged through 180 degrees of rotation at 0.7 degree increment steps. To include the full insect, four connected scans were taken in approximately 160 min. The collected images were processed through a 3D reconstruction process using NRecon and edited with CTvox software, provided by Bruker.

## 3. Results

To characterize the microbiome of *Platypus cylindrus* mycangia from the galleries of five different cork trees, we successfully amplified the bacterial 16S V4 region of all samples. After quality control and sequence filtering, a total of 234,785 reads (median: 4984) were obtained from 26 out of the initial 30 samples (14 females and 12 males; Supplementary Table S2). The reads were then grouped into 1418 ASVs with an average length of 245 bp.

### 3.1. Bacterial Community Composition

**Figure 1:**
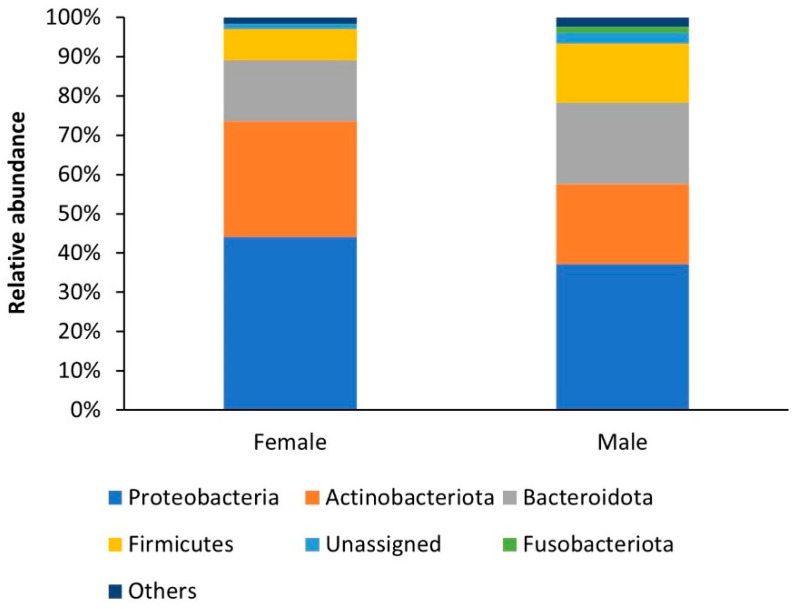
Bacterial community composition in female and male *P. cylindrus* mycangia. Bar charts show the relative abundance of bacteria at the phylum level.

A total of 26 bacterial phyla, 57 classes, 144 orders, 252 families, and 505 genera were identified in the mycangia, without removing the singletons. At high taxonomic levels, the bacterial community found in the *P. cylindrus* mycangia of female and male beetles was quite similar, although it differed in the percentage of abundance. Proteobacteria was the most abundant group in females and males (44% and 37%, respectively), followed by Actinobacteriota (29% and 20%), Bacteroidota (16% and 21%), Firmicutes (8% and 15%), and Fusobacteriota (0% and 1%) (Figure 1).

**Figure 2:**
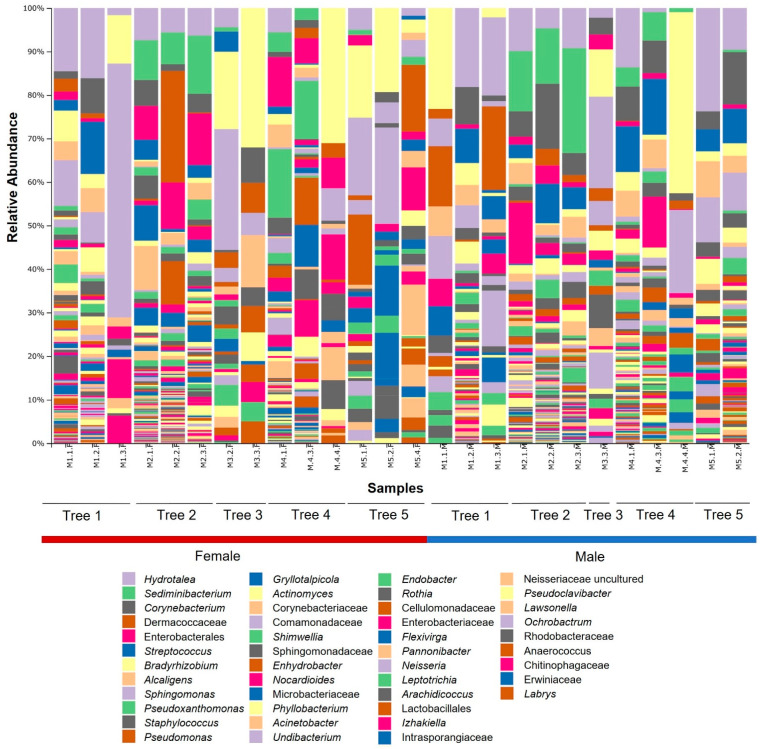
Bacterial community composition in female and male *P. cylindrus* mycangia. Bar charts show the relative abundance of bacteria at the genus level (order or family level if not further classifiable) across the 14 female and 12 male samples. Each bar corresponds to a pool of three individuals of the same sex collected from the same gallery. Only the 45 major genera (relative abundance > 0.5%) are displayed in the legend.

Most of the 45 genera of the five major phyla showing a relative abundance higher than 0.5% were present in both sexes. The most abundant bacteria both in female and male adults were *Bradyrhizobium* (3.07% and 4.3%, respectively), *Alcaligenes* (2.50% and 4.4%), *Sphingomonas* (2.36% and 2.56%) (Proteobacteria), *Corynebacterium* (2.95% and 8.58%) (Actinobacteriota), *Hydrotalea* (6.15% and 9.37%), *Sediminibacterium* (6.01% and 8.43%) (Bacteroidota), and *Streptococcus* (2.58% and 7.53%) (Firmicutes). In the mycangia of female beetles, some of the most abundant bacteria were Enterobacterales (7.53%), *Pseudoxanthomonas* (3.98%), *Pseudomonas* (3.48%), (Proteobacteria), Dermacoccaceae (8.26%), *Gryllotalpicola* (3.28%), Corynebacteriaceae (2.64%) (Actinobacteriota), and *Staphylococcus* (2.54%) (Firmicutes). In contrast to those in females, higher relative abundances in male beetle’s mycangia were instead sparsely distributed across more genera. One of the most abundant genera in male adults was *Actinomyces* (2.86%) (Actinobacteriota). Interestingly, four out of five bacteria belonging to the Enterobacterales order (i.e., Enterobacterales, *Izhakiella*, Enterobacteriaceae, Erwiniaceae) had a bigger contribution to the composition of Proteobacteria in females rather than in males (Table 1; Figure 2).

### 3.2. Core Bacteriome

The core bacteria of *P. cylindrus* mycangia present in over half of the samples of each gender were represented by 17 ASVs, corresponding to 15 taxa. Male and female mycangia shared five genera: *Alcaligens*, *Bradyrhizobium*, *Enhydrobacter*, *Hydrotalea*, and *Staphylococcus*. Furthermore, males had 10 additional taxa: *Actinomyces*, *Anaerococcus*, *Corynebacterium*, Lactobacillales, *Lawsonella*, *Ornithinimicrobium*, *Phyllobacterium*, *Rothia*, *Sediminibacterium*, and *Streptococcus*.

### 3.3. Microbial Population Diversity

Alpha rarefaction curves (RC) showed that good coverage of the overall community was achieved (Supplementary Figure S1) while ensuring a balance between female and male samples from all five cork trees. RC for Shannon estimator had leveled off, indicating that our analysis covered the quantitative biodiversity within the mycangia. Based on the observed ASV RC, we are aware that a qualitative biodiversity still exists that was not revealed for a few samples.

None of the four indices estimating alpha diversity of the bacteria from the mycangia reported differences by sex significant for Kruskal–Wallis (*p* < 0.05) (Supplementary Table S3). However, male samples showed higher values than females for observed ASVs and Faith’s PD, which are both qualitative measures of community richness. Furthermore, we found that the mycangial bacteria significantly varied in quality according to the five different trees of origin (Supplementary Table S4; observed ASVs, H = 13.84, *p* < 0.05, Kruskal–Wallis test; Faith’s PD, H = 14.89, *p* < 0.05, Kruskal–Wallis test). 

**Figure 3:**
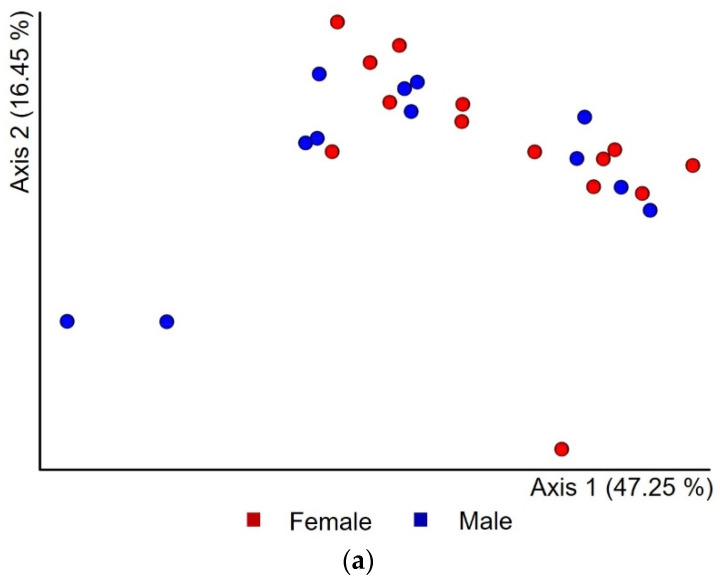

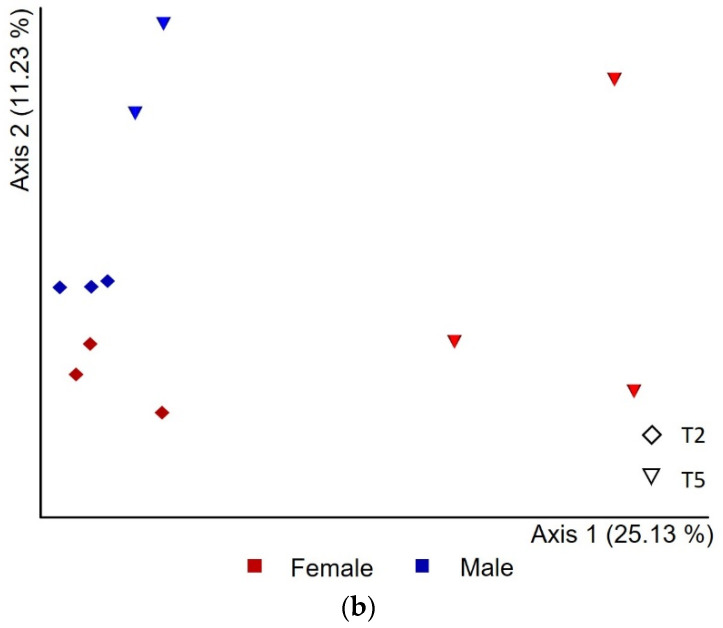
Principal coordinate analysis (PCoA) plot based on the (**a**) weighted UniFrac metric for bacterial communities. Permutational multivariate analysis of variance indicated that the bacterial community of males and females did not vary significantly (PERMANOVA’s pseudo-F = 1.80, *p* = 0.12), while the bacterial community of mycangia from different cork oaks was significantly different (PERMANOVA’s pseudo-F = 2.42, *p* = 0.01); (**b**) Bray–Curtis distance showed cluster separation between sexes in selected trees T2 and T5, without significant variation (PERMANOVA’s pseudo-F = 1.29, *p* = 0.17).

We employed different distance metrics to assess differences in bacterial community profiles between sexes and cork oak trees. PCoA based on a weighted UniFrac distance matrix (Figure 3a) and PERMANOVA revealed that the mycangia microbiota composition of *P. cylindrus* did not differ between sexes (PERMANOVA’s pseudo-F = 1.80, *p* = 0.12). However, the composition significantly differed between the five tested cork oak trees (PERMANOVA’s pseudo-F = 2.42, *p* = 0.01). Interestingly, PCoA based on a Bray–Curtis distance matrix (Figure 3b) showed a separation of the bacterial composition of female and male mycangia from cork trees T2 and T5, but not at significant levels (PERMANOVA’s pseudo-F = 1.29, *p* = 0.17). Statistical results for sex dissimilarities were consistent with those obtained from unweighted UniFrac and Jaccard (data not shown).

**Figure 4:**
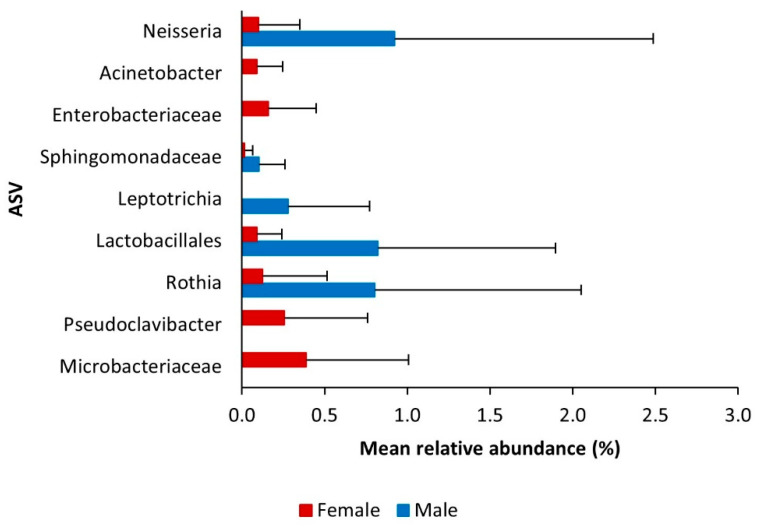
Mean relative abundance (%) of amplicon sequence variants (ASVs) classified at the genus level (order or family level if not further classifiable) and significantly differentially abundant between female and male *P. cylindrus* mycangia (Welch’s *t*-test, *p* < 0.05; Supplementary Table S6). Error bars represent standard deviation.

We further investigated whether specific genera of bacteria (with an abundance of at least 0.5%) and ASVs were statistically significant across female and male mycangia (Table 1). The relative abundances of the taxa *Pseudoclavibacter*, Chitinophagaceae, and Erwiniaceae in females were significantly higher than in males (Supplementary Table S5). In addition to these, four ASVs classified as Microbacteriaceae, *Pseudoclavibacter*, Enterobacteriaceae, and *Acinetobacter* were present only in females and showed significant differential abundance (Supplementary Table S6; Figure 4). In contrast, four taxa and five ASVs had significantly higher abundance in males: *Rothia* (Genus, ASV), Lactobacillales (Genus, ASV), *Leptotrichia* (Genus, ASV), *Neisseria* (Genus, ASV), and Sphingomonadaceae (ASV). Among them, the ASV classified as *Leptotrichia* was present only in males (Supplementary Tables S5 and S6; Figure 4). Overall, these 11 genera and ASVs showed abundances below 1.7%.

### 3.4. Anatomical Structure of Mycangia

**Figure 5:**
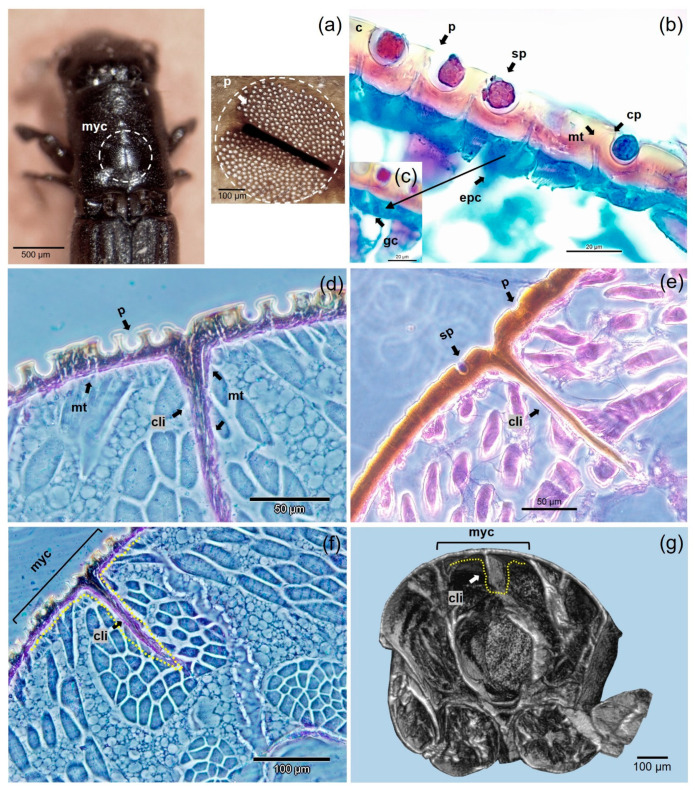
Morphological view of the prothoracic mycangia of *P. cylindrus* adult beetles: (**a**) magnification of a female mycangia with a stereo microscope (scale bars = 500 and 100 µm); 2D histological sections (**b**–**d**,**f**) of a female (scale bars = 20, 50 and 100 µm, respectively) and (**e**) of a male (scale bar = 50 µm); (**g**) 3D reconstruction of a female mycangia based on µ-CT scans (scale bar = 100 µm). Abbreviations: c, cuticle; cp, cuticular pore; cli, cuticular layer invagination; epc, epithelial cell; gc, glandular cell; myc, mycangia; mt, microtubule; p, pits; sp, spores.

The histological examination of the morphology of female and male mycangia of *P. cylindrus* adults revealed a difference in the structures connecting to the mycangial pits (Figure 5), not demonstrated before. Our histological images of female mycangia show that the pits are equipped with microtubular structures linking to glandular cells (Figure 5b,c). In Figure 5d, we observed that some microtubules extend also throughout the central invagination of the cuticular layer. In contrast, the histology of mycangial pits in males did not show microtubular structures (Figure 5e). Figure 5f,g provides a novel overall view of the transverse section of the female prothoracic mycangia with histology and 3D reconstruction. 

## 4. Discussion

This work represents the first study of the bacterial microbiota of female and male beetles of *Platypus cylindrus* and reveals important relations with their nutrition and ecological role. The bacterial community of *P. cylindrus* mycangia showed a very diverse group of bacteria belonging to different taxa, without any major one prevailing. The 45 genera with higher relative abundances were mainly shared by both females and males, and the most abundant genera accounted for less than 10% of each. This generally agrees with findings on the ambrosia beetle *Xyleborus affinis*, despite differences in beetle subfamily, host plant, artificial medium, and mycangia type [7].

Interestingly, a group of low abundance bacteria (i.e., <1.7%) corresponding to 11 genera varied significantly between females and males. This confirms the general idea provided by the available literature regarding a homogeneous community with few and specific differences. For example, a similar number of bacteria were differentially abundant in female and male beetle feces of a bark beetle species, including *Microbacterium*, *Pseudomonas*, and *Streptococcus* [9].

The presence of the core bacteriome (*Hydrotalea*, *Enhydrobacter*, *Alcaligens*, *Bradyrhizobium*, and *Staphylococcus)* in females and males might cover basic functions in the ecology of the beetles. All these bacteria were reported in the literature, associated with wood insects [9,39,40,41,42,43]. *Hydrotalea* is linked to cellulose degradation [44] and belongs to the Chitinophagaceae family, whose members are capable of degrading chitinous fungal cell walls and, therefore, are potentially involved in the control of fungi associated with *P. cylindrus*. The rare genus of *Enhydrobacter* is another bacterium with cellulolytic potential [45]. Moreover, both *Alcaligenes* and *Bradyrhizobium* are typically soil bacteria [46,47]. *Alcaligens* sp. was reported as harboring antifungal properties against both entomopathogenic and plant pathogenic fungi [47]. *Bradyrhizobium* found in Mediterranean forest soil and bark beetles [42,46] is frequently described in decaying wood, possibly connected with N-fixation [48]. This feature can benefit fungal and beetle growth within galleries, since wood is a substrate otherwise poor in available nitrogen [49].

Furthermore, bacteria of genus *Staphylococcus* are present in several habitats harboring virulence factors and potentially infecting insects [50,51], but can also be beneficial to plants by controlling pathogenic fungi [52]. *Staphylococcus* spp. can co-occur with specific fungi, which can mediate their ecological distributions. It was shown that *Penicillium* growing on cheese curd containing *Staphylococcus* promoted bacterial growth and downregulated genes associated with amino acid metabolism [50]. Interestingly, this filamentous fungus is known to inhabit the mycangia of *P. cylindrus* [53]. Therefore, the co-occurrence of such microorganisms might be ultimately related to the life quality of *P. cylindrus* within cork oak galleries.

The mycangia also harbored other bacteria such as *Sphingomonas* and *Ochrobactrum*, potentially providing food nourishment for the population of *P. cylindrus* within tree galleries. Indeed, eggs, larvae, nymphs, and adults co-exist together with diversified food requirements [10]. These bacteria were reported in adults of the ambrosia beetle *X. affinis* and were predicted to fixate nitrogen and synthesize amino acids, vitamins, and cofactors [7].

**Figure 6:**
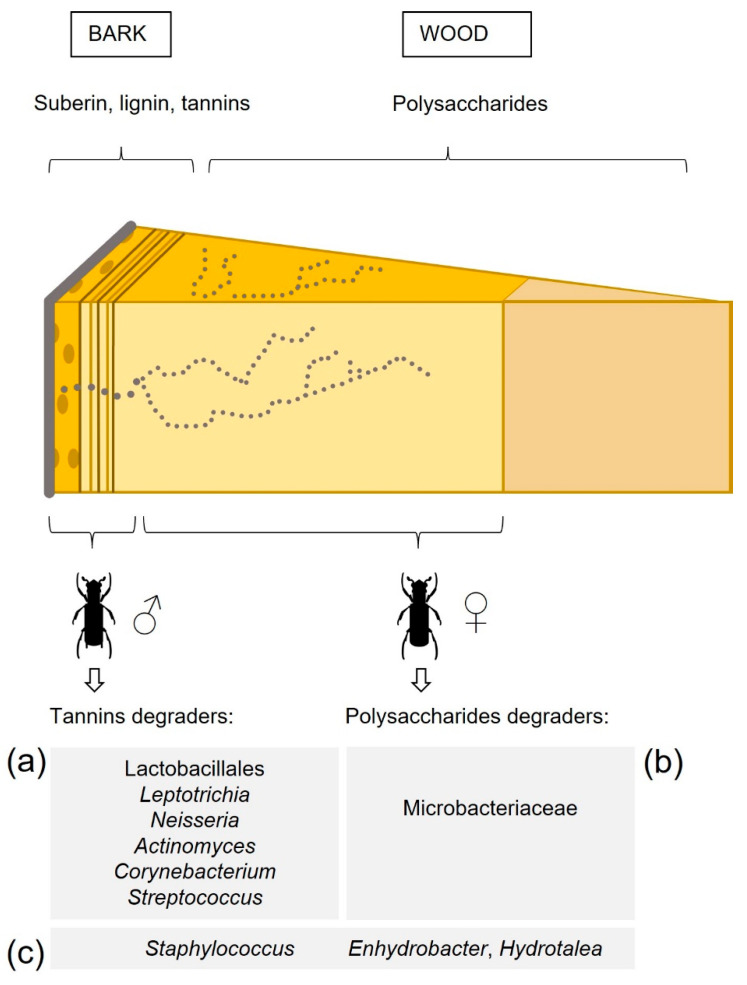
Scheme of a wood section of a *Q. suber* stem representing galleries of *P. cylindrus*, and showing the area bored by each sex. Microbiome bacterial taxa reported in the literature as tannins degraders or polysaccharides degraders are organized by having significantly higher relative abundance (Welch’s *t*-test, *p* < 0.05) and/or by belonging to the core bacteriome (**a**) of males, (**b**) of females, and (**c**) common to males and females.

One aspect of the ecological implications is the establishment by male beetles of the first penetration gallery in the trunk. The outer layers of cork oak pose a physical and chemical challenge to *P. cylindrus* and its symbionts. Cork oak stems present: (1) an outer layer of cork formed by suberin (the main cell wall component), lignin, and higher content of extractives such as tannins and lipophilic compounds, (2) the phloem, and (3) the wood. The last two are lignocellulosic tissues with moderate content of extractives. Moreover, wood has the highest content of polysaccharides [54]. In our study, we observed a pattern in the distribution of specific bacteria in male mycangia of *P. cylindrus*, according to the different wood material of the gallery’s parts built by the insects (Figure 6). For example, three out of four taxa differentially abundant in male beetles (Lactobacillales, *Leptotrichia* and *Neisseria*) were listed in a review on bacterial proteins with enzymatical tannase activity by de las Rivas et al. [55]. In addition, *Actinomyces*, *Corynebacterium*, and *Streptococcus* were also on this list and showed high relative abundance values in males and were comprised in the male core bacteriome. Other bacteria with confirmed tannase activity included *Staphylococcus* and *Sphingomonas* [55]. Tannins are plant secondary metabolites, with toxic effects on various organisms being part of the constitutive defenses deployed by oaks under biotic stress [56]. Therefore, these bacteria might be responsible for facilitating the excavation of the first tunnel made by adult male beetles and in tannin detoxification throughout the galleries. Moreover, both adult male and female beetles harbored bacterial taxa that are described in the literature as having a role in cellulose degradation; females showed high values in relative abundance for the genera *Gryllotalpicola*, *Pseudoxanthomonas* [57] and for members of the Micrococcinae suborder (e.g., Cellulomonadaceae, Microbacteriaceae; [58,59]). 

The transport of symbiotic fungi is done in specialized organs located on the prothorax as described by Cassier et al. [4], and our study confirmed its structure. The mycangium was composed of spheroidal cavities containing round spores. However, at the image resolution of this study, the wick of microtubules previously described [4] was not visible. The anatomical differences between male and female adults of *P. cylindrus* are reported by Belhoucine et al. [60], underlining traits such as mouth and maxillary palpi, and supporting a clear link between the ecological role of each sex and the body parts. More advanced structures were revealed for females, as often reported for other ambrosia beetles [61,62]. The images in this study suggest expanding this assumption to the mycangia.

In our study, the histological examination of the morphology of female and male mycangia of *P. cylindrus* adults revealed a difference in the structures connecting to the mycangial pits, not clearly demonstrated before. The histology of mycangial pits in males did not show microtubular structures. In contrast, the female mycangia showed that the pits were equipped with microtubular structures, which Cassier et al. [4] described as connections to glandular cells beneath. Some of these microtubular structures extended throughout the central invagination of the cuticular layer. How these are linked to each other remains to be confirmed. As in *P. koryoensis* [63], the cuticular pores (which lead to the glandular cells through microtubules) were present in females but not in males. Moreover, *Austroplatypus incompertus* displayed an analogous longitudinal section of the female mycangia that was equipped with microtubules and glandular cells [64]. However, variability can occur among genera of the Platypodinae subfamily [65,66].

These structural findings on the mycangia of female beetles specifically equipped with microtubules that seem extended throughout the central invagination of the cuticular layer, possibly leading to internal organs, can open new hypotheses. For example, hypotheses on translocation of microbes between internal and external organs, as well as on vertical transmission within the genus *Platypus*. Interestingly, Kudo et al. [67] observed that phylogenetically more closely related beetle species had more phylogenetically similar gut communities of Enterobacteriaceae, suggesting that these bacteria may be transmitted vertically. In our study, bacteria of the Enterobacterales order had generally high relative abundance within female samples, with two genera from families of Erwiniaceae and Enterobacteriaceae being statistically significant. Moreover, genera belonging to Enterobacterales can be either insect- or plant-pathogenic. The role of this group of bacteria in *P. cylindrus* mycangia and their interactions with cork oak requires deeper studies.

At the community level, there were no significant differences in alpha and beta diversity between female and male mycangia of *P. cylindrus*. Diversity indices suggested that it is the host tree that shapes the bacterial community and not the beetle gender. Pielou’s Evenness indicated that both sexes had a similar occurrence of the same microbiota. Slightly higher values of observed ASVs in male samples may reflect the male habit to occupy the external part of the gallery and therefore be exposed to bacteria from the environment. However, the Bray–Curtis index also indicated that in two trees, there was a small difference between the bacteria of female and male beetles. We therefore think that more trees need to be studied to clarify patterns at the community level.

## 5. Conclusions

To the best of our knowledge, this study provides the first insights into sexual differentiation for bacteria inhabiting the mycangia of a Platypodinae species. We highlighted relevant relations linking the bacteria to nutrition and other key ecological roles. Our results show that within a general homogeneous bacterial community, female and male beetles of *P. cylindrus* showed: (i) a core bacteriome potentially involved in the establishment of the beetle and its symbiotic fungi, (ii) male-specific taxa, some of which likely linked to the gallery’s portion built by the males and to the wood’s physicochemical characteristics, as well as (iii) female-specific taxa, such as some Enterobacterales. Our use of histological and 3D imaging techniques sheds light on the anatomical connections between the pits of the mycangia and the underlying tissues in females and on their absence in males, opening new hypotheses on microbial translocation between organs. Further studies are ongoing to understand the ecological role of these bacteria in the interactions with the cork oak galleries.

## Figures and Tables

**Table 1:** Shared genera between the mycangia of female and male beetles of *P. cylindrus*. Identity of the genera (order or family if not further classifiable) and their relative abundances with a minimum of 0.5% within at least one sex are displayed.

Phylum	Genus	Female Mycangia (%)		Male Mycangia (%)	
Actinobacteriota	Dermacoccaceae	8.26%		1.53%	
	*Gryllotalpicola*	3.28%		0.21%	
	*Corynebacterium*	2.95%		8.58%	
	Corynebacteriaceae	2.64%		0.42%	
	Microbacteriaceae	1.70%	‡	0.21%	
	Cellulomonadaceae	1.18%		0.08%	
	*Nocardioides*	1.10%		0.85%	
	*Flexivirga*	1.04%		0.05%	
	*Actinomyces*	0.81%		2.86%	
	*Pseudoclavibacter*	0.77%	†/‡	0.05%	
	*Lawsonella*	0.56%		0.28%	
	Intrasporangiaceae	0.43%		0.52%	
	*Rothia*	0.26%		1.24%	†/‡
Bacteroidota	*Hydrotalea*	6.15%		9.37%	
	*Sediminibacterium*	6.01%		8.43%	
	*Arachidicoccus*	0.85%		0.15%	
	Chitinophagaceae	0.64%	†	0.09%	
Firmicutes	*Staphylococcus*	2.54%		1.72%	
	*Streptococcus*	2.48%		7.53%	
	*Anaerococcus*	0.25%		0.58%	
	Lactobacillales	0.16%		0.98%	†/‡
Fusobacteriota	*Leptotrichia*	0.02%		1.17%	†/‡
Proteobacteria	Enterobacterales	7.53%		1.69%	
	*Pseudoxanthomonas*	3.98%		0.45%	
	*Pseudomonas*	3.48%		0.35%	
	*Bradyrhizobium*	3.07%		4.30%	
	*Alcaligenes*	2.50%		4.14%	
	*Sphingomonas*	2.36%		2.56%	
	Comamonadaceae	1.40%		1.77%	
	*Shimwellia*	1.38%		1.74%	
	Neisseriaceae uncultured	1.20%		0.77%	
	Sphingomonadaceae	1.02%		1.69%	‡
	*Endobacter*	0.97%		0.46%	
	*Izhakiella*	0.92%		0.04%	
	*Phyllobacterium*	0.81%		0.87%	
	Enterobacteriaceae	0.77%	‡	0.45%	
	Erwiniaceae	0.67%	†	0.04%	
	*Enhydrobacter*	0.66%		1.84%	
	*Acinetobacter*	0.63%	‡	1.04%	
	*Undibacterium*	0.58%		0.97%	
	*Labrys*	0.52%		0.00%	
	*Pannonibacter*	0.49%		0.63%	
	*Ochrobactrum*	0.35%		0.50%	
	Rhodobacteraceae	0.30%		0.55%	
	*Neisseria*	0.14%		1.05%	†/‡

Significance in differential abundance (Welch’s *t*-test, *p* < 0.05) of bacteria between sexes is marked with the symbol (†) for taxa and with the symbol (‡) for ASVs (statistical results in Supplementary Tables S5 and S6).

## Data Availability

We confirm that the raw data supporting the findings of this study are openly available at the links https://www.ebi.ac.uk/ena/browser/view/PRJEB44517 (accessed on 28 May 2021) and http://doi.org/10.5281/zenodo.4899899 [68].

## Supplementary Materials

Supplementary material associated with this article can be found in the online version at https://www.mdpi.com/article/10.3390/insects12100881/s1. Figure S1: Alpha rarefaction curves of the 26 mycangia samples based on high-throughput sequencing (Illumina MiSeq) of bacterial communities: (a) Shannon, (b) observed ASVs, (c) number of samples by sex, and (d) number of samples by cork oak tree. Color-coded lines represent all the samples from mycangia; Table S1: DAtest results of the statistical test comparisons: (a) sequences collapsed at the genus level, (b) ASVs; Table S2: Bacteria 16S rRNA gene sequences and samples after quality filtering and rarefaction of input data; Table S3: Alpha diversity indices for mycangia of female and male adult beetles; Table S4: Alpha diversity indices for mycangia of beetles from different cork oaks; Table S5: Statistical results of the differential abundant analysis of the bacterial genera in female and male mycangia of *P. cylindrus* for Welch’s *t*-test (*p* < 0.05); Table S6: Statistical results of the differential abundant analysis of the bacterial sequences (ASVs) in female and male mycangia of *P. cylindrus* for Welch’s *t*-test (*p* < 0.05).
