# Supplementary material for: Microbiome Associated with the Mycangia of Female and Male Adults of the Ambrosia Beetle Platypus cylindrus Fab. (Coleoptera: Curculionidae)
Source: Insects. 2021 Sep 29;12(10):881. doi: 10.3390/insects12100881 (PMC8540956; doi:10.3390/insects12100881)
Supplement: Supplementary file 1 [file insects-12-00881-s001.zip › Supplementary Figure S1.pdf]

Figure S1 Alpha rarefaction curves of the 26 mycangia samples based on high-throughput sequencing (Illumina MiSeq) of bacterial communities: (a) Shannon, (b) observed ASVs, (c) number of samples by sex and (d) number of samples by cork oak tree. Color-coded lines represent all the samples from mycangia.

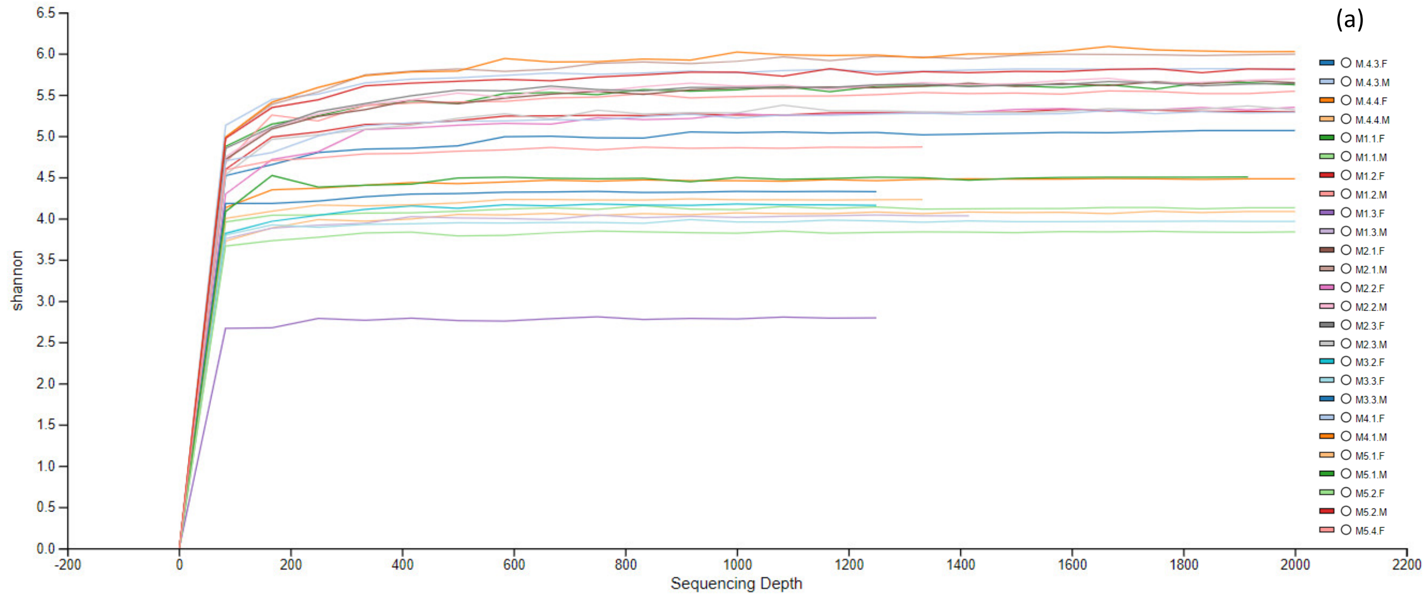

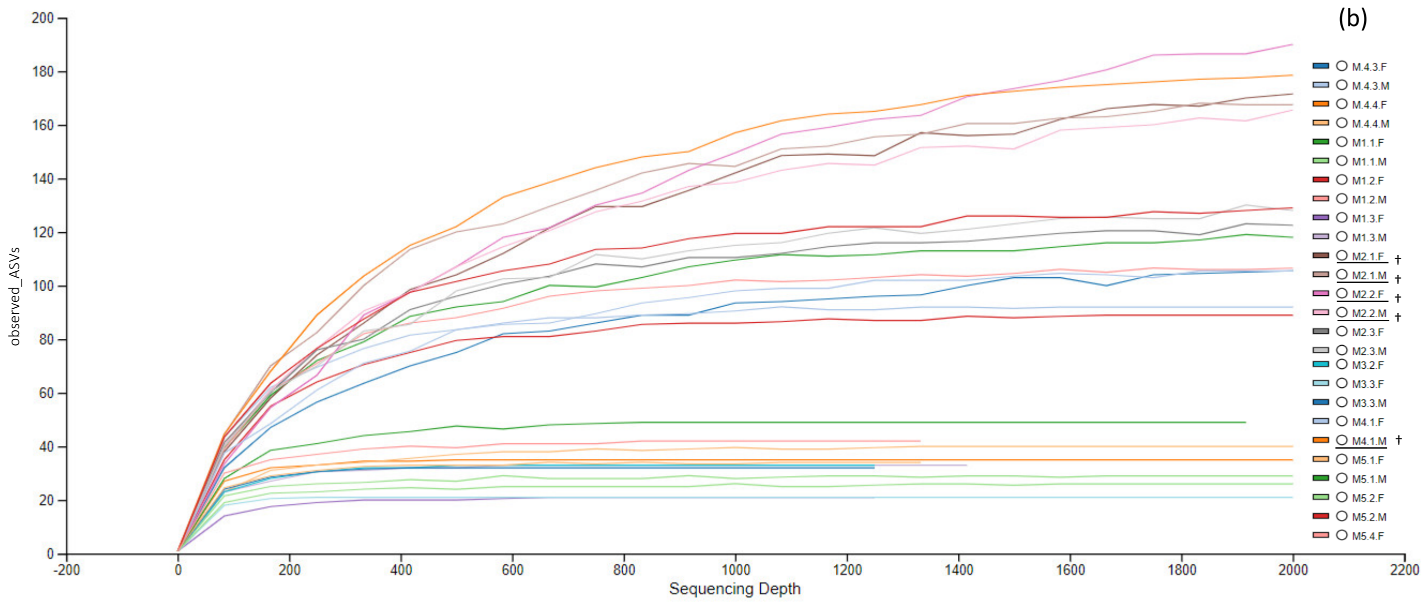

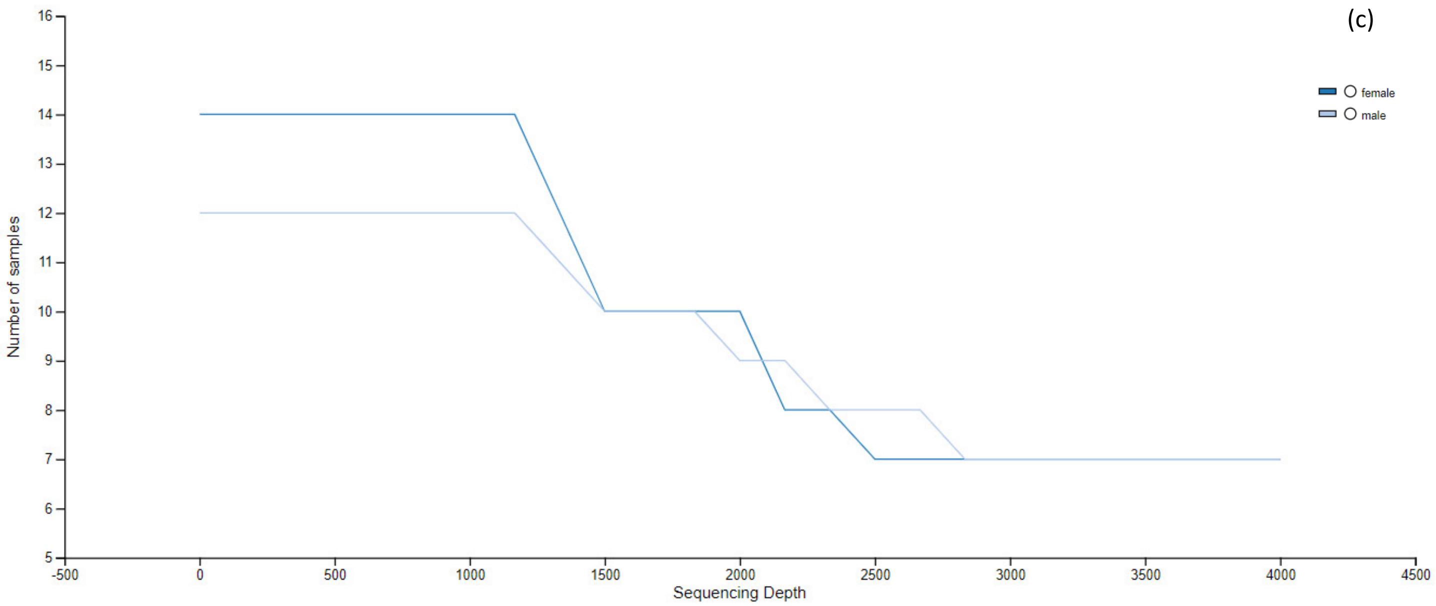

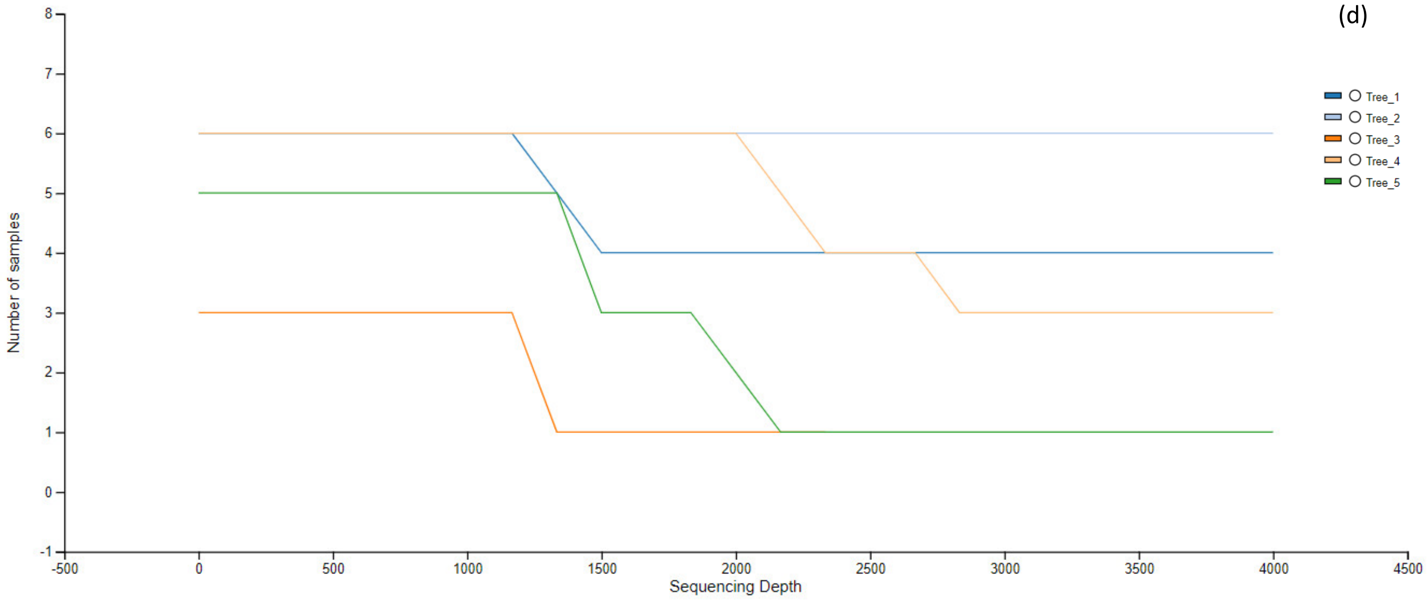

† Underscores in plot (b) highlight samples with observed ASVs ≥ 160 at 2000 sequencing depth

The 26 samples are distributed across female and male mycangia of five trees: six in Tree_1 (Three females and three males; M1.1F, M1.1M, M1.2F, M1.2M, M1.3F, M1.3M), six in Tree_2 (Three females and three males; M2.2M, M2.3F, M2.3M, M2.1F, M2.1M, M2.2F), three in Tree_3 (Two females and one male; M3.2F, M3.3F, M3.3M), six in Tree_4 (Three females and three males; M4.1F, M4.1M, M4.3F, M4.3M, M4.4F, M4.4M) and five in Tree_5 (Three females and two males; M5.1F, M5.1M, M5.2F, M5.2M, M5.4F)
