# Supplementary material for: Microbiome Associated with the Mycangia of Female and Male Adults of the Ambrosia Beetle Platypus cylindrus Fab. (Coleoptera: Curculionidae)
Source: Insects. 2021 Sep 29;12(10):881. doi: 10.3390/insects12100881 (PMC8540956; doi:10.3390/insects12100881)
Supplement: Supplementary file 1 [file insects-12-00881-s001.zip › Supplementary Tables S1-S6.pdf]

Table S1 DAtest results of the statistical test comparisons: (a) sequences collapsed at genus level, (b) ASVs

(a)

Method	AUC	FPR	FDR	Power	Score	Score.5%	Score.95%	
Log t-test (ltt)	0.90	0.04	0.00	0.23	0.09	0.00	0.27	*
Welch's t-test - CLR (ttc)	0.93	0.05	0.00	0.20	0.09	-0.08	0.22	*
Permutation (per)	0.75	0.04	0.00	0.20	0.05	-0.24	0.10	*
Wilcox (wil)	0.92	0.03	0.00	0.07	0.03	0.00	0.20	*
t-test - ALR (tta)	0.89	0.03	0.00	0.07	0.03	-1.02	0.25	*
Log t-test2 (ltt2)	0.86	0.03	0.00	0.00	0.00	0.00	0.17	*
t-test (ttt)	0.77	0.01	0.00	0.00	0.00	0.00	0.00	*
Quasi-Poisson GLM (qpo)	0.60	0.13	0.38	0.07	-0.37	-1.00	0.04	
Poisson GLM (poi)	0.73	0.81	0.93	0.93	-0.71	-0.85	-0.58	
Negbinom GLM (neb)	0.70	0.36	0.88	0.67	-0.75	-0.84	-0.49	

(b)

Method	AUC	FPR	FDR	Power	Score	Score.5%	Score.95%	
Welch's t-test - CLR (ttc)	0.93	0.03	0.00	0.19	0.08	0.00	0.31	*
Log t-test (ltt)	0.92	0.03	0.00	0.08	0.04	0.00	0.21	*
Permutation (per)	0.67	0.04	0.00	0.17	0.03	-0.06	0.07	*
Wilcox (wil)	0.94	0.02	0.00	0.00	0.00	0.00	0.13	*
Log t-test2 (ltt2)	0.85	0.03	0.00	0.00	0.00	0.00	0.05	*
t-test (ttt)	0.75	0.01	0.00	0.00	0.00	0.00	0.00	*
t-test - ALR (tta)	0.94	0.01	0.00	0.00	0.00	-1.00	0.26	*
Quasi-Poisson GLM (qpo)	0.61	0.14	0.25	0.00	-0.25	-1.00	0.00	
Poisson GLM (poi)	0.72	0.80	0.93	0.92	-0.73	-0.87	-0.70	
Negbinom GLM (neb)	0.67	0.36	0.90	0.61	-0.79	-0.94	-0.69	

"Area Under the (Receiver Operator) Curve" (AUC), "False Positive Rate" (FPR), "False Discovery Rate" (FDR) and "Empirical power" (Power).
Asterisks (*) in the summary output means that a method is equally good as the best method.

Table S2 Bacteria 16S rRNA gene sequences and samples after quality filtering and rarefaction of input data

No. of samples	Sample-id	Sample-ref	Denoised	Merged	Non-chimeric	Bacterial sequences	Rarefied
**1**	sample-22	M.4.3.M	123956	109180	108690	2678	1260
**2**	sample-7	M2.1.F	53847	51806	51044	31579	1260
**3**	sample-9	M2.2.F	47336	45272	43723	38282	1260
**4**	sample-20	M4.1.M	34305	32898	32238	29297	1260
**5**	sample-21	M.4.3.F	31524	30500	30386	13750	1260
**6**	sample-10	M2.2.M	28401	26910	25712	22744	1260
**7**	sample-12	M2.3.M	21907	20588	20303	14028	1260
**8**	sample-11	M2.3.F	15520	14955	14773	8475	1260
**9**	sample-19	M4.1.F	13862	13633	13496	9951	1260
**10**	sample-28	M5.2.M	12867	12442	12310	11284	1260
**11**	sample-4	M1.2.M	13119	12253	12073	6726	1260
**12**	sample-8	M2.1.M	11760	10855	10440	8272	1260
**13**	sample-1	M1.1.F	10522	10014	9893	8767	1260
**14**	sample-3	M1.2.F	7317	6879	6777	5329	1260
**15**	sample-2	M1.1.M	4982	4786	4749	4639	1260
**16**	sample-17	M3.3.F	2809	2764	2734	2453	1260
**17**	sample-24	M.4.4.M	2684	2479	2458	2255	1260
**18**	sample-23	M.4.4.F	2681	2394	2356	2138	1260
**19**	sample-26	M5.1.M	2405	2264	2232	1963	1260
**20**	sample-27	M5.2.F	2279	2224	2214	2116	1260
**21**	sample-18	M3.3.M	2008	1957	1953	1260	1260
**22**	sample-25	M5.1.F	1584	1524	1511	1361	1260
**23**	sample-6	M1.3.M	1585	1522	1502	1490	1260
**24**	sample-29	M5.4.F	2613	1488	1479	1354	1260
**25**	sample-15	M3.2.F	1498	1426	1417	1332	1260
**26**	sample-5	M1.3.F	1318	1284	1275	1262	1260
27	sample-14	M3.1.M	1292	1265	1265	984	
28	sample-13	M3.1.F	1256	1181	1164	1105	
29	sample-16	M.3.2.M	827	805	777		
30	sample-30	M.5.4.M	600	547	544		

Table S3 Alpha diversity indices for mycangia of female and male adult beetles

Alpha-diversity index	Gender		Kruskal-Wallis test	
	female	male	H	*p*
**Observed_ASVs**	73.43	91.75	1.11	0.291
**Shannon**	4.71	5.09	2.22	0.136
**Pielou_e**	0.81	0.82	1.42E-14	1
**Faith_pd**	9.15	14.95	1.17	0.28

The sequences were rarefied to 1260 sequences. Mean values for n= 14 (female) and n=12 (male)

Table S4 Alpha diversity indices for mycangia of beetles from different cork oaks

**Alpha-diversity index**	**Cork oak**					**Kruskal-Wallis test**	
	**Tree_1**	**Tree_2**	**Tree_3**	**Tree_4**	**Tree_5**	**H**	***p***
**Observed_ASVs**	64.83	142.5	28.67	87.33	55	13.84	0.008
**Shannon**	4.59	5.56	4.15	5.08	4.64	7.54	0.11
**Pielou_e**	0.8	0.78	0.87	0.82	0.84	7.06	0.133
**Faith_pd**	6.39	22.55	3.71	13.92	7.83	14.89	0.005

The sequences were rarefied to 1260 sequences. Mean values for n= 6 (Tree_1), n=6 (Tree_2), n=3 (Tree_3), n=6 (Tree_4) and n=5 (Tree_5)

Table S5 Statistical results of the differential abundant analysis of the bacterial genera in female and male mycangia of *P. cylindrus* for Welch's t-test ($p < 0.05$)

Taxonomy	*p*	log2FC	ordering	Genus	Method
d__Bacteria; p__Fusobacteriota; c__Fusobacteriia; o__Fusobacteriales; f__Leptotrichiaceae; g__Leptotrichia	0.003	2.22	male>female	*Leptotrichia*	Welch's t-test - CLR (ttc)
d__Bacteria; p__Bacteroidota; c__Bacteroidia; o__Chitinophagales; f__Chitinophagaceae;__	0.006	-1.86	female>male	Chitinophagaceae	Welch's t-test - CLR (ttc)
d__Bacteria; p__Actinobacteriota; c__Actinobacteria; o__Micrococcales; f__Micrococcaceae; g__Rothia	0.015	1.84	male>female	*Rothia*	Welch's t-test - CLR (ttc)
d__Bacteria; p__Proteobacteria; c__Gammaproteobacteria; o__Enterobacterales; f__Erwiniaceae;__	0.037	-1.42	female>male	Erwiniaceae	Welch's t-test - CLR (ttc)
d__Bacteria; p__Firmicutes; c__Bacilli; o__Lactobacillales;__;__	0.039	1.39	male>female	Lactobacillales	Welch's t-test - CLR (ttc)
d__Bacteria; p__Actinobacteriota; c__Actinobacteria; o__Micrococcales; f__Microbacteriaceae; g__Pseudoclavibacter	0.040	-1.49	female>male	*Pseudoclavibacter*	Welch's t-test - CLR (ttc)
d__Bacteria; p__Proteobacteria; c__Gammaproteobacteria; o__Burkholderiales; f__Neisseriaceae; g__Neisseria	0.046	1.45	male>female	*Neisseria*	Welch's t-test - CLR (ttc)

Table S6 Statistical results of the differential abundant analysis of the bacterial sequences (ASVs) in female and male mycangia of *P. cylindrus* for Welch's t-test ($p < 0.05$)

ASVs	*p*	log2FC	ordering	Genus	Method
1b1c565a3cafb2fed30c8bcea39a77c9	0.001	-2.05	female>male	Microbacteriaceae	Welch's t-test - CLR (ttc)
2bd9309f2f97cae51d18d06ea1ca519a	0.009	1.90	male>female	Lactobacillales	Welch's t-test - CLR (ttc)
d83f60183d81253a505beaeef3cd168f	0.013	1.91	male>female	*Rothia*	Welch's t-test - CLR (ttc)
929c82d69b2f15cffe7adf61b8c81fc8	0.015	-1.30	female>male	*Pseudoclavibacter*	Welch's t-test - CLR (ttc)
4d72007c70f4abc0bf9e8fe8b826d07d	0.025	-1.21	female>male	Enterobacteriaceae	Welch's t-test - CLR (ttc)
b6825773b8b549f2ab996dcd37e1bedd	0.026	1.34	male>female	*Leptotrichia*	Welch's t-test - CLR (ttc)
dd36571922ab7a138ee06438ab9c7891	0.031	1.71	male>female	*Neisseria*	Welch's t-test - CLR (ttc)
947540e2f4a0221b97ceb93c35936ad4	0.033	1.01	male>female	Sphingomonadaceae	Welch's t-test - CLR (ttc)
c22b16cc6108c04f29fea3b6d4c81571	0.047	-1.05	female>male	*Acinetobacter*	Welch's t-test - CLR (ttc)
